# Supplementary material for: Treatment, toxicity, and mortality after subsequent breast cancer in female survivors of childhood cancer
Source: Nat Commun. 2025 Mar 31;16:3088. doi: 10.1038/s41467-025-58434-w (PMC11958683; doi:10.1038/s41467-025-58434-w)
Supplement: Supplementary file 1 — Supplementary Information [file 41467_2025_58434_MOESM1_ESM.pdf]

SUPPLEMENTARY FIGURES

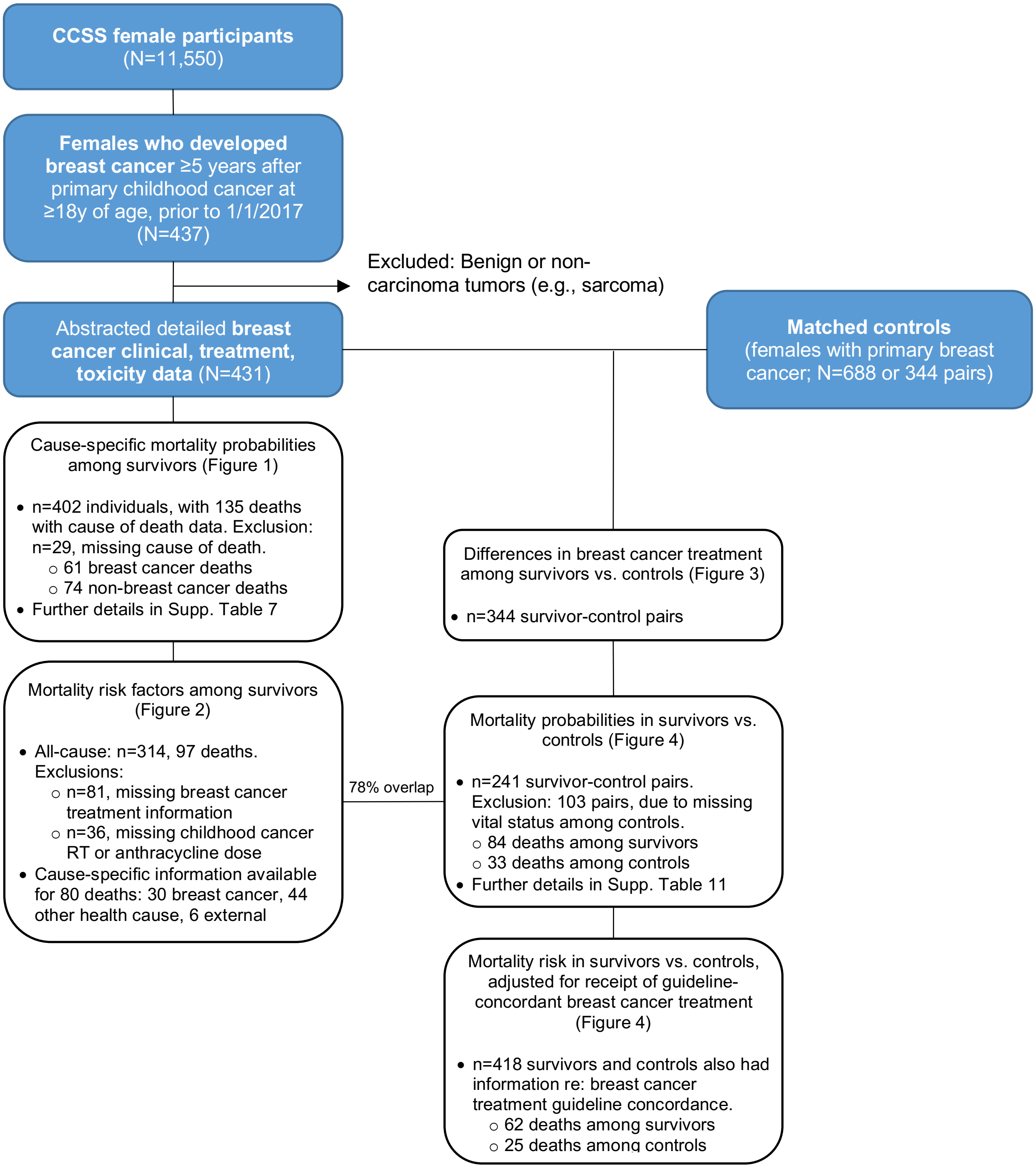

Supplementary Figure 1: Overview of participant inclusions and exclusions across all primary analyses.

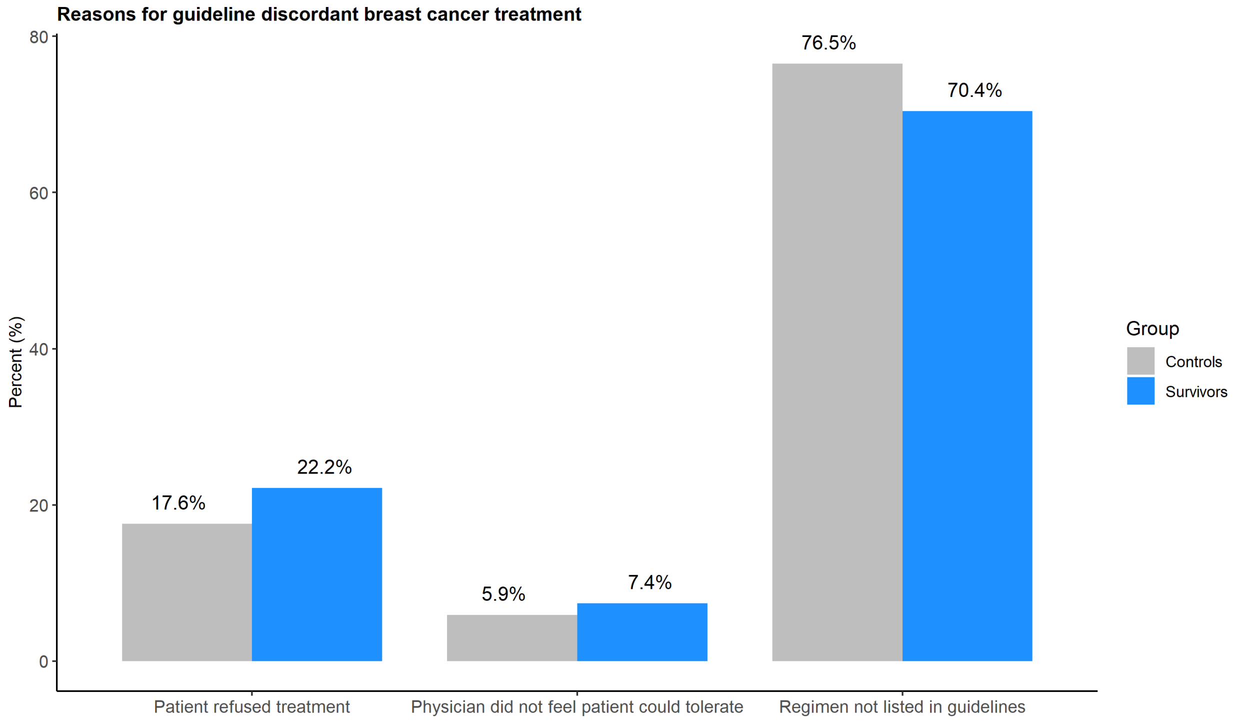

Supplementary Figure 2: Reasons for breast cancer guideline discordant treatment among survivors (n=27) and controls (n=17).

SUPPLEMENTARY TABLES

Supplementary Table 1: Non-breast subsequent malignant neoplasms (SMNs) diagnosed before and after breast cancer among childhood cancer survivors.

**Non-BC other SMN type**	**Pre BC**	**Post BC**
Acute myeloid leukemia	0	3
Female genital	1	2
Lung and bronchus	1	8
Lymphoid leukemia	1	1
Non-Hodgkin lymphoma	2	3
Other cancers	4	5
Other head and neck	0	2
Other soft tissue sarcomas	0	4
Parotid gland	2	1
Renal neoplasms	2	0
Skin	3	1
Small intestine and colorectal	1	4
Thyroid	10	5
Unspecified soft tissue sarcomas	0	3
**Total**	27	42

Abbreviations: ALL, acute lymphoblastic leukemia; AML, acute myeloid leukemia; BC, breast cancer; CCSS, Childhood Cancer Survivor Study; NHL, Non-Hodgkin lymphoma; NMSC, nonmelanoma skin cancer; SMN, subsequent malignant neoplasm.
Note: 58 CCSS survivors experienced a non-NMSC SMN before or after breast cancer. Above table includes multiple SMNs per survivor. Within this group, primary diagnosis: Hodgkin lymphoma (41); osteosarcoma (6), Ewing sarcoma (4), soft tissue sarcoma (2), ALL (1), AML (1), NHL (1), other leukemia (1), neuroblastoma (1).

Supplementary Table 2: Primary cancer and breast cancer characteristics among childhood cancer survivors by primary cancer diagnosis group

	HL (N=251)		Leukemia (N=47)		Sarcoma (N=84)		NHL (N=18)		Other (N=31)	
Characteristic	N	% or median (IQR)	N	% or median (IQR)	N	% or median (IQR)	N	% or median (IQR)	N	% or median (IQR)
Age at childhood cancer diagnosis (years)	251	16 (14-18)	47	12 (10-14)	84	15 (12-16)	18	14 (11-18)	31	7 (4-12)
Race										
White	242	96.4	44	94.0	79	94.0	17	94.4	30	96.8
Non-white	9	3.6	3	6.0	5	6.0	1	5.6	1	3.2
Treated with chest radiotherapy for childhood cancer[a]	226	96.6	12	25.2	18	22.5	9	56.3	14	48.3
Received anthracyclines for childhood cancer[b]	61	25.9	29	65.1	62	75.6	13	76.5	9	30.0
Age at first breast cancer diagnosis(years)	251	40 (35-45)	47	40 (35-43)	84	40 (34-44)	18	39 (35-42)	31	38 (33-43)
Years between primary and breast cancer diagnosis	251	23 (18-29)	47	26 (23-32)	84	25 (19-29)	18	24 (18-30)	31	28 (22-35)
Breast cancer treatment era										
Before 2000	80	31.9	4	8.1	19	22.6	6	33.3	6	19.4
2000-2009	105	41.8	26	52.4	40	47.6	4	22.2	11	35.5
2010-2019	66	26.3	17	39.6	25	29.8	8	44.4	14	45.2
Breast cancer histology[c]										
DCIS/LCIS	66	26.5	5	10.3	20	24.1	0	0	10	32.3
Invasive	183	73.5	41	89.7	63	75.9	18	100	21	67.7
Vital status										
Dead	111	44.2	11	22.2	29	34.5	5	27.8	8	25.8

Abbreviations: HL, Hodgkin lymphoma; NHL, Non-Hodgkin lymphoma; IQR, interquartile range; DCIS/LCIS, ductal carcinoma in situ/lobular carcinoma in situ.

a. Missing chest radiotherapy information (HL: 17; leukemia: 2; sarcoma: 4; NHL: 2; other: 2)
b. Missing anthracycline information (HL: 15; leukemia: 1; sarcoma: 2; NHL: 1; other: 1)
c. Missing breast cancer histology (HL: 2; leukemia: 1; sarcoma: 1)

Supplementary Table 3: Breast cancer treatments among CCSS survivors only

	All survivors (N=431)		Chest RT for primary (N=279)		No chest RT for primary (N=125)	
Breast cancer treatment	N	% or median (IQR)	N	% or median (IQR)	N	% or median (IQR)
Prescribed guideline-concordant breast cancer treatment[a]						
No	27	7.5	16	7.0	10	9.4
Yes	329	92.5	214	93.0	94	90.6
Surgery[b]						
Underwent surgery for breast cancer	410	98.1	271	99.3	116	95.96
Bilateral mastectomy	246	60.3	184	67.9	47	41.84
Treatment groups[c]						
Mastectomy without RT	258	69.4	189	77.5	53	50.29
Lumpectomy with RT	45	12.0	13	5.3	29	26.21
Neither	70	18.6	42	17.2	26	23.5
Hormone modulating therapy						
Among ER+[d]						
Any SERM	146	54.4	89	53.3	51	58.2
Any AI	80	29.8	53	31.7	23	26.24
Any SERM or AI	178	66.3	111	66.5	59	67.32
HER2+ therapy						
Among HER2+[e]						
Any HER2-targeted therapy	39	60.9	20	52.6	15	71.43
Radiation therapy (RT)[f]						
Received RT	99	25.7	41	16.5	52	45.36
Cumulative prescribed dose[g]						
Median (IQR), Grays	79	59 (50-60)	31	50 (48-60)	43	60 (52-60)
Chemotherapy[h]						
Received any chemotherapy	208	53.8	132	52.0	63	57.26
Cyclophosphamide	139	67.2	90	68.2	42	68.01
Any taxanes	136	65.8	85	64.4	44	71.05
Any anthracyclines	96	46.8	64	48.5	28	46.68
Fluorouracil	55	26.1	33	25.0	16	24.38
Methotrexate	33	15.7	21	15.9	8	12.19
Carboplatin	27	12.8	17	12.9	7	10.66
Cyclophosphamide/doxorubicin regimens[i]						
Cyclophosphamide, doxorubicin	76	37.3	56	42.4	19	32.96
Cyclophosphamide, doxorubicin, and taxane	45	22.6	35	26.5	10	19.25
Cyclophosphamide, doxorubicin, and no taxane	31	14.7	21	15.9	9	13.71

Abbreviations: BC, breast cancer; RT, radiation therapy; HMT, hormone modulating therapy; AI, aromatase inhibitor; SERM, selective estrogen receptor modulator; HER2, human epidermal growth factor receptor 2.

a. Missing information in entire sample (n=75) and chest RT-only (n=49) and no chest RT subgroups (n=21).
b. Missing surgery information in entire sample (n=13) and chest RT-only (n=6) and no chest RT subgroups (n=4). Bilateral mastectomy proportion only among those treated with surgery for breast cancer only.
c. Missing information in entire sample (n=37) and chest RT-only (n=27) and no chest RT subgroups (n=8). Mastectomy refers to unilateral or bilateral mastectomy.
d. ER+ disease only, in entire sample (n=266) and chest RT-only (n=167) and no chest RT subgroups (n=85).
e. HER+ disease only, in entire sample (n=64) and chest RT-only (n=38) and no chest RT subgroups (n=21).
f. RT information missing in entire sample (n=48) and chest RT-only (n=31) and no chest RT subgroups (n=13).
g. RT dose information missing in entire sample (n=20) and chest RT-only (n=10) and no chest RT subgroups (n=9).
h. Chemotherapy information missing in entire sample (n=42) and chest RT-only (n=25) and no chest RT subgroups (n=13).
i. Among those treated with chemotherapy only.

Supplementary Table 4: Risk factors influencing breast cancer treatment decisions in childhood cancer survivors.

Treatment decision	Variables	OR (95% CI)	P
Prescribed guideline-discordant treatment (n=329, 27 with guideline-discordant treatment)			
	Invasive breast cancer histology	11.62 (1.52-88.98)	0.018
	Prior subsequent malignant neoplasm	2.85 (0.53-15.27)	0.22
	Prior major cardiovascular event	1.16 (0.31-4.38)	0.83
	Any chest RT for primary cancer	1.25 (0.48-3.28)	0.64
	Any anthracyclines for primary cancer	4.18 (1.48-11.85)	7.1×10^{-3}
Not treated with radiotherapy (n=343, 257 not treated)			
	Invasive breast cancer histology	0.24 (0.11-0.55)	6.8×10^{-4}
	Chest RT for childhood cancer[a]		
	Any to 35 Gy	2.35 (1.20-4.57)	0.012
	>35 Gy	4.87 (2.26-10.50)	5.3×10^{-5}
	Anthracyclines for childhood cancer[b]		
	Any to 250 mg/m^2	0.85 (0.35-2.08)	0.72
	>250 mg/m^2	0.77 (0.38-1.54)	0.46
	Prior subsequent malignant neoplasm	2.09 (0.44-9.98)	0.36
	Prior major cardiovascular event	2.39 (0.85-6.75)	0.10
Not treated with anthracyclines (n=350, 263 not treated)			
	Invasive breast cancer histology	0.10 (0.03-0.29)	2.2×10^{-5}
	Chest RT for childhood cancer[a]		
	Any to 35 Gy	1.52 (0.72-3.21)	0.27
	>35 Gy	2.05 (0.96-4.38)	0.064
	Anthracyclines for childhood cancer[b]		
	Any to 250 mg/m^2	3.06 (1.20-7.77)	0.019
	>250 mg/m^2	5.29 (2.29-12.20)	9.3×10^{-5}
	Prior subsequent malignant neoplasm	4.66 (0.57-37.73)	0.15
	Prior major cardiovascular event	2.96 (1.05-8.33)	0.040

Abbreviations: RT, radiation therapy; Gy, Gray; mg, milligrams; m, meters. Multivariable logistic regression models included covariates above and were also adjusted for age at breast cancer diagnosis and breast cancer diagnosis year. P-values are based on two-sided tests.

a. No chest RT is the referent.
b. No anthracyclines is the referent.

Supplementary Table 5: Breast cancer treatment decisions among CCSS survivors with prior non-breast subsequent malignancies or history of major cardiovascular events

Variables	N	Mastectomy without RT	Lumpectomy with RT	Neither
Prior subsequent malignant neoplasm	21	76.2 (16)	0	23.8 (5)
Prior major cardiovascular event	44	79.6 (35)	0	20.5 (9)

Supplementary Table 6: Vital status by breast cancer stage among CCSS survivors

	All survivors (N=431)		LCIS/DCIS (N=101)		Stage I/II (N=232)		Stage III/IV (N=48)	
	N	%	N	%	N	%	N	%
Vital status								
Alive	267	62.2	74	73.27	155	67.18	16	33.33
Dead	164	37.8	27	26.73	77	32.82	32	66.67
Cause	N=135		N=24		N=59		N=27	
Breast cancer	61	45.2	4	16.67	25	42.37	19	70.37
Other SN	26	19.3	6	25.00	11	18.64	2	7.41
Recurrence	8	5.9	3	12.50	4	6.78	0	0
Cardiovascular	20	14.8	5	20.83	11	18.64	2	7.41
Other health condition	11	8.2	2	8.33	5	8.47	2	7.41
External	9	6.7	4	16.67	3	5.08	2	7.41
	Lumpectomy with radiotherapy (N=45)		Mastectomy* without radiotherapy (N=258)		ER+ disease, no HMT (N=88)		ER+ disease, received HMT (N=178)	
	N	%	N	%	N	%	N	%
Vital status								
Alive	34	75.56	171	66.62	63	72.4	120	67.4
Dead	11	24.44	87	33.38	25	27.6	58	32.6
Cause	N=8		N=68		N=19		N=43	
Breast cancer	3	37.50	21	30.88	11	57.9	17	39.5
Other SN	3	37.50	14	20.59	3	15.8	12	27.9
Recurrence	0	0.00	6	8.82	2	10.5	3	7.0
Cardiovascular	2	25.00	14	20.59	1	5.3	5	11.6
Other health condition	0	0.00	7	10.29	0	0.0	5	11.6
External	0	0.00	6	8.82	2	10.5	1	2.3

Abbreviations: SN, subsequent neoplasm; DCIS/LCIS, ductal carcinoma in situ/lobular carcinoma in situ; ER+, estrogen receptor positive; HMT, hormone modulating therapy. *Mastectomy refers to unilateral or bilateral mastectomy.

Supplementary Table 7: Breast cancer- and other cause-specific mortality probabilities among CCSS survivors, stratified by disease histology, guideline-concordant breast cancer treatment receipt, and treatment modality

			5 years		10 years		15 years	
			Breast cancer	Other cause	Breast cancer	Other cause	Breast cancer	Other cause
	N	Deaths	Probability (95% CI)					
Overall	402	BC: 61<br>Other: 74	0.08 (0.06-0.12)	0.03 (0.02-0.05)	0.14 (0.11-0.18)	0.11 (0.08-0.15)	0.19 (0.15-0.24)	0.19 (0.15-0.25)
DCIS/LCIS	98	BC: 4<br>Other: 20	0.01 (0.00-0.07)	0.03 (0.01-0.10)	0.03 (0.01-0.10)	0.14 (0.08-0.24)	0.05 (0.02-0.14)	0.22 (0.14-0.36)
Invasive	300	BC: 57<br>Other: 53	0.11 (0.08-0.15)	0.03 (0.01-0.05)	0.17 (0.13-0.22)	0.10 (0.07-0.14)	0.23 (0.18-0.29)	0.18 (0.14-0.25)
Guideline-concordant care	310	BC: 32<br>Other: 51	0.04 (0.02-0.07)	0.03 (0.02-0.06)	0.09 (0.06-0.13)	0.11 (0.08-0.15)	0.14 (0.10-0.20)	0.17 (0.13-0.24)
Guideline-discordant care	23	BC: 9<br>Other: 4	0.30 (0.16-0.56)	0	0.35 (0.20-0.62)	0.16 (0.06-0.45)	0.43 (0.26-0.73)	0.26 (0.11-0.63)
Lumpectomy, RT	42	BC: 3<br>Other: 5	0	0	0.07 (0.02-0.26)	0.07 (0.02-0.29)	0.12 (0.04-0.37)	0.25 (0.11-0.56)
Mastectomy, no RT	239	BC: 21<br>Other: 47	0.03 (0.01-0.06)	0.02 (0.01-0.05)	0.07 (0.04-0.12)	0.11 (0.08-0.17)	0.12 (0.08-0.18)	0.19 (0.14-0.26)
Neither	84	BC: 19<br>Other: 15	0.19 (0.12-0.3)	0.05 (0.02-0.13)	0.24 (0.16-0.36)	0.11 (0.06-0.21)	0.24 (0.16-0.36)	0.20 (0.12-0.34)

Abbreviations: DCIS/LCIS, ductal carcinoma in situ/lobular carcinoma in situ; BC, breast cancer; RT, radiation therapy. Mastectomy refers to unilateral or bilateral mastectomy.

Supplementary Table 8: Characteristics among CCSS survivors and general population controls

	Survivors (N=344)		General population (N=344)	
	N	% or median (IQR)	N	% or median (IQR)
Age at first breast cancer diagnosis				
Median (IQR)	344	41 (36-45)	344	42 (37-47)
Treatment year				
Before 2000	63	18.2	56	16.3
2000-2009	157	45.3	146	42.4
2010-2016	124	36.5	142	41.3
Race[a]				
White	327	95.1	237	78.0
Non-White	17	4.9	67	22.0
Black	13	3.8	48	15.8
Asian/Pacific Islander	2	0.6	13	4.3
Other	2	0.6	6	2.0
Stage of first breast cancer[b]				
0	81	24.1	81	24.0
I	111	33.1	113	33.4
II	100	30.6	99	29.3
III	31	9.2	36	10.7
IV	10	3.0	9	2.7
Histology				
DCIS/LCIS only	85	24.5	85	24.7
Invasive	259	75.5	259	75.3
Progesterone receptor status[c]				
Positive	197	69.2	180	65.5
Estrogen receptor status[c]				
Positive	233	78.6	209	75.2
HER2 status[c]				
Positive	59	25.9	57	24.1
Vital status[d]				
Alive	228	66.5	208	86.3
Dead	116	33.5	33	13.7

Abbreviations: IQR, interquartile range; DCIS/LCIS, ductal carcinoma in situ/lobular carcinoma in situ; HER2, human epidermal growth factor receptor 2.

a. Missing race in 40 controls.
b. Missing breast cancer stage in 11 survivors and 6 controls.
c. Missing breast cancer hormone receptor status and HER2 status, as follows: progesterone receptor status (62 survivors, 69 controls), estrogen receptor status (47 survivors, 66 controls); HER2 status (119 survivors, 107 controls).
d. Missing vital status for 103 controls.

Supplementary Table 9: Breast cancer treatments among CCSS survivors and matched controls

	Survivors (N=344)		General population (N=344)					
Treatment modality	N	%	N	%	OR	LL	UL	P-value
Prescribed guideline-concordant breast cancer treatment[a]	291	92.7	281	94.3	1.23	0.59	2.56	0.58
Treated with radiation therapy[b]	89	27.6	188	61.4	0.23	0.15	0.34	$2.5x10^{-12}$
Treated with surgery for breast cancer[c]	336	99.1	311	97.8	2.33	0.60	9.02	0.22
Surgical procedures[d]								
Mastectomy	272	81.1	185	59.5	2.71	1.87	3.93	$1.5x10^{-7}$
Bilateral mastectomy	207	61.9	73	23.5	5.92	3.87	9.04	$2.0x10^{-15}$
Lumpectomy	101	29.8	148	47.6	0.42	0.29	0.60	$2.4x10^{-6}$
Breast cancer treatment groups[e]								
Mastectomy without radiotherapy	213	65.8	108	32.4	4.12	2.82	6.03	$2.9x10^{-13}$
Lumpectomy with radiotherapy	42	12.8	122	36.6	0.27	0.18	0.41	$4.5x10^{-10}$
Neither of these groups	70	21.4	103	30.9	0.59	0.40	0.87	$8.3x10^{-3}$
Hormone modulating therapy[f]								
Any SERM	131	55.6	142	67.9	0.45	0.27	0.75	$2.1x10^{-3}$
Any AI	74	31.4	75	35.9	0.80	0.51	1.27	0.35
Any SERM or AI	161	68.3	164	78.4	0.42	0.23	0.79	$7.2x10^{-3}$
HER2-targeted therapy[g]	36	61.02	37	64.91	1.60	0.52	4.89	0.41
Chemotherapy[h]								
Yes	176	54.5	172	55.5	0.81	0.48	1.36	0.43
Cyclophosphamide-doxorubicin regimens[i]								
Cyclophosphamide, doxorubicin	66	38.4	104	60.5	0.25	0.14	0.47	$1.1x10^{-5}$
Cyclophosphamide, doxorubicin, taxane	41	24.4	72	41.9	0.35	0.19	0.63	$4.4x10^{-4}$
Received cyclophosphamide	117	67.0	130	75.6	0.50	0.27	0.93	0.028
Any taxanes[i]	125	71.5	109	63.4	2.33	1.07	5.09	0.033
Received docetaxel	73	40.9	38	22.1	4.75	2.22	10.18	$6.2x10^{-5}$
Received paclitaxel	55	32.3	72	41.9	0.49	0.27	0.87	0.015
Any anthracyclines[i]	82	47.4	114	66.3	0.33	0.18	0.58	$1.6x10^{-4}$
Received doxorubicin	74	42.9	110	64.0	0.27	0.14	0.49	$2.1x10^{-5}$
Received fluorouracil[i]	42	23.5	15	8.7	13.00	3.09	54.77	$4.7x10^{-4}$
Received methotrexate[i]	22	12.3	8	4.7	5.00	1.45	17.27	0.011
Received carboplatin[i]	24	13.4	14	8.1	1.78	0.79	4.02	0.17

Abbreviations: OR, odds ratio; LL, 95% confidence interval lower limit; UL, 95% confidence interval upper limit; AI, aromatase inhibitor; SERM, selective estrogen receptor modulator; HER2, human epidermal growth factor receptor 2. ORs, 95% CIs and p-values (based on two-sided tests) were obtained from univariate conditional logistic regression models comparing the odds of prescribed treatments for survivors with controls.

a. Missing information for 30 survivors and 46 controls.
b. Missing information for 24 survivors and 38 controls.
c. Missing information for 5 survivors and 26 controls.
d. Among patients who were treated with surgery for breast cancer. Mastectomy refers to unilateral or bilateral mastectomy unless otherwise specified.
e. Missing information for 19 survivors and 11 controls. Mastectomy refers to unilateral or bilateral mastectomy.
f. Among 233 survivors and 209 controls with estrogen receptor-positive disease.
g. Among 59 survivors and 57 controls with HER2-positive disease.
h. Missing information among 19 survivors and 34 controls.
i. Among patients who were treated with chemotherapy.

Supplementary Table 10: Breast cancer treatment modifications or toxicities among survivors and controls treated with chemotherapy and prescribed guideline-concordant treatment

	Controls (N=111)		Survivors (N=102)			
Characteristic	Total	% (n)	Total	% (n)	OR (95% CI)	P
Any treatment modification	66	74.2% (49)	96	76.7% (73)	1.68 (0.73-3.89)	0.22
Drug omission	74	8.1% (6)	101	21.2% (22)	4.59 (1.48-14.28)	8.4×10^{-3}
Cycle omission	74	8.1% (6)	100	10.7% (11)	2.05 (0.60-7.03)	0.25
Received GCSF	66	63.6% (42)	98	64.2% (62)	1.81 (0.84-3.9)	0.13
Delay in treatment	66	16.7% (11)	97	13.0% (13)	0.71 (0.28-1.83)	0.48
Any toxicity	89	74.2% (66)	99	86.2% (85)	2.54 (1.10-5.88)	0.029
Fever and neutropenia hospitalization	81	8.6% (7)	97	17.1% (17)	2.29 (0.82-6.36)	0.11
Infection	78	11.5% (9)	97	8.0% (8)	0.56 (0.20-1.60)	0.28
Surgical complication	99	19.2% (19)	101	32.5% (31)	1.80 (0.90-3.59)	0.10
Cytopenia[a]	111	8.1% (9)	102	20.1% (21)	2.95 (1.21-7.21)	0.017
Neurotoxicity[b]	111	17.1% (19)	102	29.6% (31)	2.14 (1.04-4.37)	0.038
Cardiotoxicity	111	3.6% (4)	102	11.5% (12)	3.04 (0.85-10.92)	0.088
Pulmonary toxicity	111	3.6% (4)	102	3.8% (4)	0.75 (0.18-3.14)	0.69
Gastrointestinal toxicity	111	25.2% (28)	102	37.3% (39)	1.73 (0.92-3.27)	0.092
Dermatological toxicity	111	18.9% (21)	102	22.0% (23)	1.58 (0.74-3.37)	0.24
Musculoskeletal toxicity	111	21.6% (24)	102	17.2% (18)	0.83 (0.4-1.75)	0.63

Abbreviations: OR, odds ratio; CI, confidence interval; P, p-value; GCSF, granulocyte colony-stimulating factor. ORs, 95% CIs and p-values (based on two-sided tests) were obtained from logistic regression models comparing the odds of treatment modification or toxicity characteristics for survivors with controls, adjusting for race/ethnicity and breast cancer diagnosis age, year, and histology.

a. Includes anemia, neutropenia, and thrombocytopenia.
b. Includes peripheral neuropathy.

Supplementary Table 11: All-cause mortality probabilities in CCSS survivors and matched controls, stratified by breast cancer histology and treatment modality

	Entire sample		LCIS/DCIS		Invasive	
	Controls (n=241, 33 deaths)	Survivors (n=241, 84 deaths)	Controls (n=63, 3 deaths)	Survivors (n=63, 16 deaths)	Controls (n=178, 30 deaths)	Survivors (n=178, 68 deaths)
5-year	0.06 (0.03-0.09)	0.13 (0.08-0.17)	0.02 (0.00-0.05)	0.03 (0.00-0.08)	0.08 (0.04-0.12)	0.16 (0.10-0.21)
10-year	0.08 (0.05-0.11)	0.29 (0.22-0.35)	0.02 (0.00-0.05)	0.17 (0.05-0.27)	0.10 (0.06-0.15)	0.33 (0.25-0.41)
15-year	0.14 (0.09-0.19)	0.41 (0.32-0.49)	0.02 (0.00-0.05)	0.39 (0.19-0.54)	0.18 (0.11-0.25)	0.42 (0.32-0.50)
	Mastectomy*, no RT		Lumpectomy, RT		Neither treatment group	
	Controls (n=80, 4 deaths)	Survivors (n=147, 44 deaths)	Controls (n=94, 7 deaths)	Survivors (n=29, 8 deaths)	Controls (n=61, 20 deaths)	Survivors (n=53, 23 deaths)
5-year	0.02 (0.00-0.06)	0.05 (0.02-0.09)	0.01 (0.00-0.03)	0.04 (0.00-0.11)	0.20 (0.09-0.29)	0.28 (0.14-0.39)
10-year	0.02 (0.00-0.06)	0.23 (0.15-0.30)	0.02 (0.00-0.05)	0.20 (0.00-0.35)	0.23 (0.12-0.33)	0.43 (0.26-0.56)
15-year	0.02 (0.00-0.06)	0.32 (0.21-0.41)	0.08 (0.01-0.14)	0.49 (0.16-0.69)	0.39 (0.22-0.52)	0.51 (0.31-0.66)

Abbreviations: DCIS/LCIS, ductal carcinoma in situ/lobular carcinoma in situ; RT, radiation therapy. *Mastectomy refers to unilateral or bilateral mastectomy.

Supplementary Table 12: All-cause mortality hazard ratios comparing survivors to matched controls, adjusting for key matching variables

Subgroup	HR (95% CI)	P
Entire sample	4.14 (2.44-7.00)	$1.2x10^{-7}$
LCIS/DCIS	9.77 (2.04-46.79)	$4.3x10^{-3}$
Invasive	3.45 (1.96-6.07)	$1.8x10^{-5}$
Mastectomy, no radiotherapy	14.70 (3.58-60.42)	$1.9x10^{-4}$
Lumpectomy, with radiotherapy	7.77 (2.00-30.20)	$3.1x10^{-3}$
Neither treatment group	1.73 (0.84-3.55)	0.14

Abbreviations: DCIS/LCIS, ductal carcinoma in situ/lobular carcinoma in situ; HR, hazard ratio; CI, confidence interval; P, p-value. All models adjusted for receipt of guideline-discordant care and the following matching variables: first breast cancer diagnosis age and year; race/ethnicity; breast cancer histology (except in LCIS/DCIS and invasive subgroups). Mastectomy refers to unilateral or bilateral mastectomy. P-values are based on two-sided tests.

Supplementary Table 13: Risk factors for all-cause mortality among survivors and controls with invasive breast cancer (N=211)

Variables	HR (95% CI)	P
Survivor (vs. control)	2.06 (1.01-4.18)	0.046
Developed ≥1 acute toxicity (versus none)	0.92 (0.41-2.10)	0.85
Had ≥1 treatment modification (versus none)	1.45 (0.68-3.10)	0.34

All survivors and controls were treated with chemotherapy and were prescribed guideline-concordant treatment for breast cancer. Multivariable Cox proportional hazards regression model was adjusted for first breast cancer diagnosis age and year and race/ethnicity. P-values are based on two-sided tests.

Supplementary Table 14: Overall observed (crude) survival probabilities in all females with first primary breast cancer diagnosed between 1981-2016 at ages 35-49 years with stage information in SEER

	Matched controls (N=238)	SEER (N=98,488)		
Stage	Stage % (N)	Stage % (N)	5-year observed survival (95% CI)	10-year observed survival (95% CI)
0	25.6% (61)	21.9% (21,563)	99.0 (98.9-99.2)	97.4 (97.1-97.6)
I-IV	74.4% (177)	78.1% (76,925)	88.7 (88.4-88.9)	81.4 (81.1-81.7)
I	45.8% (81)	40.8% (31,350)	97.2 (97.0-97.4)	93.6 (93.3-93.8)
II	33.9% (60)	39.4% (30,280)	91.3 (91.0-91.7)	84.0 (83.5-84.4)
III	16.4% (29)	16.2% (12,494)	73.1 (72.3-73.9)	58.9 (58.0-59.8)
IV	4.0% (7)	3.6% (2,801)	32.7 (30.9-34.4)	17.1 (15.7-18.6)

SEER database: Incidence - SEER Research Data, 8 Registries, November 2022 (1975-2020). Missing stage information for 3 controls with invasive breast cancer.
