# Supplementary material for: Treatment, toxicity, and mortality after subsequent breast cancer in female survivors of childhood cancer
Source: Nat Commun. 2025 Mar 31;16:3088. doi: 10.1038/s41467-025-58434-w (PMC11958683; doi:10.1038/s41467-025-58434-w)
Supplement: Supplementary file 2 — Reporting Summary [file 41467_2025_58434_MOESM2_ESM.pdf]

nature portfolio

Corresponding author(s): Lucie M. Turcotte
Last updated by author(s): Mar 3, 2025

Reporting Summary

Nature Portfolio wishes to improve the reproducibility of the work that we publish. This form provides structure for consistency and transparency in reporting. For further information on Nature Portfolio policies, see our Editorial Policies and the Editorial Policy Checklist.

Statistics

For all statistical analyses, confirm that the following items are present in the figure legend, table legend, main text, or Methods section.

n/a	Confirmed	
☐	☒	The exact sample size (*n*) for each experimental group/condition, given as a discrete number and unit of measurement
☐	☒	A statement on whether measurements were taken from distinct samples or whether the same sample was measured repeatedly
☐	☒	The statistical test(s) used AND whether they are one- or two-sided<br>*Only common tests should be described solely by name; describe more complex techniques in the Methods section.*
☐	☒	A description of all covariates tested
☐	☒	A description of any assumptions or corrections, such as tests of normality and adjustment for multiple comparisons
☐	☒	A full description of the statistical parameters including central tendency (e.g. means) or other basic estimates (e.g. regression coefficient) AND variation (e.g. standard deviation) or associated estimates of uncertainty (e.g. confidence intervals)
☐	☒	For null hypothesis testing, the test statistic (e.g. *F*, *t*, *r*) with confidence intervals, effect sizes, degrees of freedom and *P* value noted<br>*Give P values as exact values whenever suitable.*
☒	☐	For Bayesian analysis, information on the choice of priors and Markov chain Monte Carlo settings
☒	☐	For hierarchical and complex designs, identification of the appropriate level for tests and full reporting of outcomes
☒	☐	Estimates of effect sizes (e.g. Cohen's *d*, Pearson's *r*), indicating how they were calculated

Our web collection on statistics for biologists contains articles on many of the points above.

Software and code

Policy information about availability of computer code

Data collection	Please see the code availability statement in our manuscript.
Data analysis	Please see the code availability statement in our manuscript.

For manuscripts utilizing custom algorithms or software that are central to the research but not yet described in published literature, software must be made available to editors and reviewers. We strongly encourage code deposition in a community repository (e.g. GitHub). See the Nature Portfolio guidelines for submitting code & software for further information.

Data

Policy information about availability of data

All manuscripts must include a data availability statement. This statement should provide the following information, where applicable:

- Accession codes, unique identifiers, or web links for publicly available datasets
- A description of any restrictions on data availability
- For clinical datasets or third party data, please ensure that the statement adheres to our policy

Please see the data availability statement in our manuscript.

Human research participants

Policy information about studies involving human research participants and Sex and Gender in Research.

Reporting on sex and gender	Analyses were limited to females only. Gender information was not considered as a part of this analysis. Sex was considered in the design of this study and sex is based self-/proxy-report or information reported from the treating institution.
Population characteristics	Female survivors with a confirmed breast cancer diagnosis (ductal carcinoma in situ [DCIS], lobular carcinoma in situ [LCIS], or invasive carcinoma) as of December 31, 2016 at age ≥18 years and ≥5 years after initial childhood cancer diagnosis were included
Recruitment	This was a retrospective observational study--all available female survivors of childhood cancer with a subsequent breast cancer diagnosis from the Childhood Cancer Survivor Study were included in the analysis; matched controls were identified from registries at three large academic medical centers (Duke University, University of Chicago, University of Minnesota) based on tumor characteristics, year of diagnosis, race and ethnicity
Ethics oversight	This study was approved by the University of Minnesota IRB, as well as at the University of Chicago and Duke University, and the overall Childhood Cancer Survivor Study was reviewed and approved by the IRBs of all participating institutions

Note that full information on the approval of the study protocol must also be provided in the manuscript.

Field-specific reporting

Please select the one below that is the best fit for your research. If you are not sure, read the appropriate sections before making your selection.

☒ Life sciences ☐ Behavioural & social sciences ☐ Ecological, evolutionary & environmental sciences

For a reference copy of the document with all sections, see nature.com/documents/nr-reporting-summary-flat.pdf

Life sciences study design

All studies must disclose on these points even when the disclosure is negative.

Sample size	All females with subsequent breast cancer diagnosis (invasive ductal carcinoma or in situ carcinoma) within the Childhood Cancer Survivor Study were included in the analyses; the control group was based on 1:1 matching with the subsequent breast cancer cases.
Data exclusions	For analyses describing treatment or toxicity, we excluded cases or controls that did not have treatment or toxicity data available
Replication	No replication was done--this was a retrospective observational study
Randomization	No randomization was done--this was a retrospective observational study
Blinding	No blinding was done--this was a retrospective observational study

Reporting for specific materials, systems and methods

We require information from authors about some types of materials, experimental systems and methods used in many studies. Here, indicate whether each material, system or method listed is relevant to your study. If you are not sure if a list item applies to your research, read the appropriate section before selecting a response.

Materials & experimental systems

n/a	Involved in the study	
☒	☐	Antibodies
☒	☐	Eukaryotic cell lines
☒	☐	Palaeontology and archaeology
☒	☐	Animals and other organisms
☒	☐	Clinical data
☒	☐	Dual use research of concern

Methods

n/a	Involved in the study	
☒	☐	ChIP-seq
☒	☐	Flow cytometry
☒	☐	MRI-based neuroimaging
